# Alteration of eggs biochemical composition and progeny survival by maternal high carbohydrate nutrition in a teleost fish

**DOI:** 10.1038/s41598-022-21185-5

**Published:** 2022-10-06

**Authors:** Therese Callet, Emilie Cardona, Nicolas Turonnet, Patrick Maunas, Laurence Larroquet, Anne Surget, Genevieve Corraze, Stephane Panserat, Lucie Marandel

**Affiliations:** INRAE, Universite de Pau et des Pays de l’Adour, E2S UPPA, NUMEA, 64310 Saint-Pee-sur-Nivelle, France

**Keywords:** Physiology, Metabolism

## Abstract

Reproductive performances, and the factors affecting them, are of major importance especially for farmed fish in the context of the development of a sustainable aquaculture. Dietary maternal lipids have been identified as a major factor affecting reproductive performances. Nevertheless, the consequences of carbohydrates have been little studied while plant-derived carbohydrates could be increasingly used in broodstock diets. To explore this issue, 2-year-old female trout were fed either a control diet that contains no carbohydrate and a high protein content (65.7%) or a diet formulated with plant-derived carbohydrates containing 32.5% carbohydrate and 42.9% protein (’HC diet’) for an entire reproductive cycle. The reproductive performances, the quality of the unfertilized eggs and the development of the progeny were carefully monitored. Although the one year HC nutrition had not impaired female growth nor spawns quality, such nutrition had increased the variability of eggs size within spawns (+ 34.0%). Moreover, the eggs produced had a modified fatty acid profile, including a significant reduction in EPA content (− 22.9%) and a significant increase in the AA/EPA ratio (+ 33.3%). The progeny were impacted by such alterations as their survival rates were significantly reduced. A lower plant-derived carbohydrate inclusion (20%) should be considered in aquafeed for female broodstock in trout.

## Introduction

Reproductive performance traits have been extensively studied, in particular to determine the factors affecting them. Regarding teleost fish, high reproductive performances encompass both the number of eggs produced per female (absolute fecundity and relative fecundity when expressed per units of weight) and the proportion of high quality eggs within spawn^1^. High quality eggs are eggs that present high fertilization rate, high survivability at the different critical stage (eyeing, hatching and first feeding) and that generate viable offspring^1,2^. The intrinsic characteristics of the egg itself, namely its genes, its maternal mRNA transcripts and the composition of the yolk, determine its quality^1,2^. Embryos rely on egg yolk to perform their developments until first feeding^3^. Thus, yolk composition that derives from maternal sources (nutrients and hormones are incorporated during the growth of the oocytes), directly influences progeny development^4^. An adequate nutrition of female broodstock is thus recognized as one of the most important determining factors to maintain high reproductive performances and to produce high quality gametes^5^.

The relationship between the maternal nutrition and reproduction has been particularly explored and more specifically on farmed species that have an economic importance^1^. On that subject, a particular attention was paid to lipid sources and to the consequences of unbalanced fatty acids (FA) profiles in broodstock diet^5^. Content of essential fatty acid is considered as the most important nutritional factors affecting reproductive performance in fish^5^. In contrast, concerning species that belong to higher trophic levels, because broodstock do not have specific requirement for dietary carbohydrates^6^, far less studies have been dedicated to this nutrient. Nevertheless, to promote sustainability of aquaculture production, plant-derived carbohydrates must be increasingly added in their diets to replace the traditional protein-rich fishmeal^7^. Then, the issue of carbohydrates in broodstock aquafeed should also be considered.

Only two studies in rainbow trout have explored the consequences of the lowering of the protein-to-carbohydrate ratio in maternal diet^8,9^. The first study has demonstrated that lowering protein-to-carbohydrate ratio in female broodstock ratio surprisingly increased relative fecundity, hatching rate and survival of their progeny at the eyed stage, but the factors contributing to this improvement have not been identified^8^. On the opposite, the most recent study calls into question some of these results. Although reproductive performances were maintained and the biochemical composition of the eggs was not affected, the survival of the progeny was high but not improved^9^. In the latter, the number of females used for the experiment was limited while a massive variability in reproductive performances is usually observed within trout female populations^10^, hiding some potential effects.

The present study aims to deepen the exploration of the consequences of a maternal high carbohydrate nutrition on their reproductive performances but also on the embryo and fry development. An understanding of the consequences of such a maternal diet is absolutely necessary before increasing the proportion of plant-based carbohydrates in farming practice. To do so, 3-year old female trout were fed either a control diet formulated with fishmeal that contains no carbohydrate (named ’control diet’) or a diet formulated with plant-derived carbohydrates containing 32.5% carbohydrate, namely ’HC diet’, for an entire reproductive cycle. In order to have two isoenergetic experimental diets, the ‘HC diet’ had a lower protein content (42.9%), which is still in line with known nutritional requirements. The reproductive performances, the quality of the unfertilized eggs, determined with a new and robust phenotypical tool^10^ and the development of the progeny (at the embryo and at the yolk-sac fry stages) were carefully monitored.

## Results

### Female Broodstock growth

**Figure 1:**
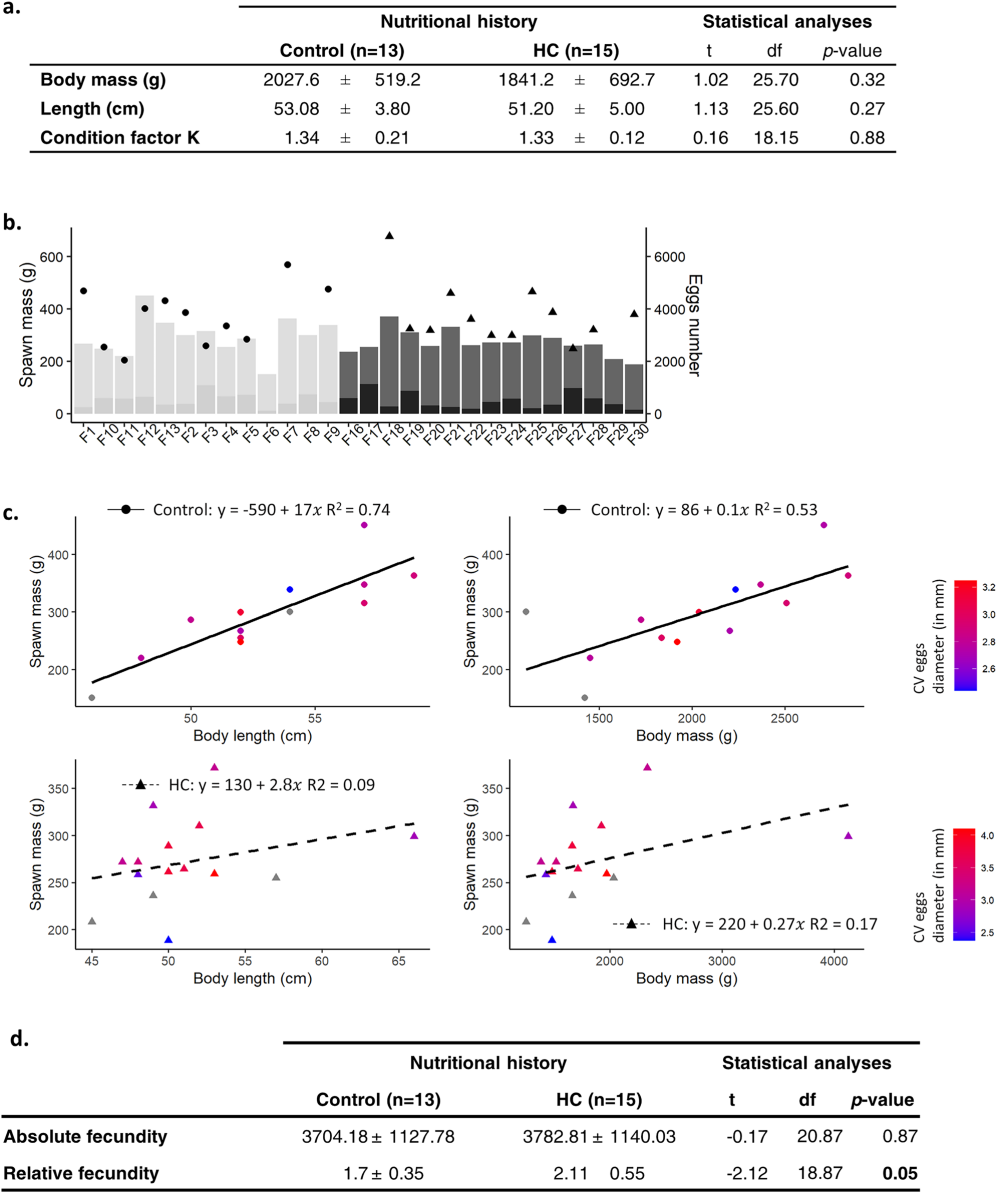
Reproductive performances. (**a**) Growth performances of females fed the control diet or the high carbohydrate diet (HC diet) after the 11-month nutrition trial. (**b**) Total spawning mass, which includes coelomic fluid mass and egg mass, of ’control spawn’ (i.e. spawn produced by females fed the control diet) in light grey and ’HC spawn’ (i.e. spawn produced by females fed the high carbohydrate diet) in dark grey and estimated number of eggs in each ’control spawn’ (circle) and in each ’HC spawn’ (triangle). (**c**) Correlation between the spawn mass and the female total length and total mass. The control females are represented by circles (solid line) and the ’HC’ ones by triangles (dashed line), which are coloured according to the value of the coefficient of variability (CV) of eggs diameter. Spawns with a low CV in eggs diameter are coloured in blue while spawns with large CV are coloured in red. (**d**) The average absolute fecundity (number of eggs produced by female) and the relative fecundity (number of eggs produced per unit of weight) of females fed the control diet and the HC diet (in bold, the significant differences between the two condition).

After the 11-month nutrition trial, spawns obtained from 13 control females and 15 females fed the ’HC’ diet (namely ’HC females’) were sampled. Broodstock female fed the ’HC’ diet had a similar mean body mass, length and K index as females fed the control diet (Fig. 1a).

### Reproductive performances: spawn characteristics

To assess the effects of the maternal HC nutrition on reproductive performances, spawns produced by the control females and ’HC females’ were weighted and analysed in order to control egg viability. Spawns produced by ’HC females’ had the same mass (total, coelomic fluid and eggs mass) than spawns produced by the control females (Fig. 1b). For the control females, the spawn mass was strongly correlated with their total length (R^2^ = 0.74, p-value < 0.001) and their total mass (R^2^ = 0.54, p-value = 0.005). Such correlation was not found for the females fed the HC diet (R^2^ = 0.09 and R^2^ = 0.17 for length and mass, respectively, p-values > 0.05, Fig. 1c). The average absolute fecundity (number of eggs produced by female) of ’HC females’ was similar to the one of control females. The average relative fecundity (number of eggs produced per unit of female weight) was significantly increased in ’HC’ females (+ 24.1%, p-value = 0.05, Fig. 1d). With the exception of one ’HC spawn’ containing 25.1% of non-viable eggs, all other produced spawns were of high quality as less than 2.5% of the eggs they contained were non-viable, regardless of the maternal diet.

### Unfertilized eggs characteristics

Regarding inter-spawn variability, the surface of the eggs produced was significantly different from one spawns to another, independent of the maternal nutritional history (p-value < 0.001). But, in average, females fed the HC diet produced eggs which had a similar surface to those produced by the control females (p-value = 0.26, Table 1). In average, the coefficient of variation (CV) of ’HC eggs’ surface (i.e. eggs produced by females fed the HC diet) was significantly higher than ’control eggs’ (p-value = 0.02, Table 1).

**Table 1:** Eggs characteristics.

	Nutritional history		Statistical analyses		
	Control (n = 2999)	HC *(n* = *3710)*	F	df	*p*-value
Eggs surface (mm^2^)	21.29 ± 2.95	20.08 ± 2.79	1.34	1.00	0.26

	Nutritional history		Statistical analyses		
	Control (n = 11)	HC *(n* = *12)*	t	df	*p*-value
Coefficient variation (surface)	**6.47 ± 0.98**	**8.67 ± 2.76**	**− 2.59**	**13.97**	**0.02**

	Nutritional history		Statistical analyses		
	Control (n = 13)	HC *(n* = *15)*	t	df	*p*-value
**Proportion**					
Dry matter (DM, %)	**36.09 ± 1.15**	**37.38 ± 1.36**	**− 2.72**	**26.00**	**0.01**
Protein content (%)	25.71 ± 1.17	26.48 ± 1.03	− 1.82	24.02	0.08
Lipid content (%)	**9.69 ± 0.58**	**10.10 ± 0.47**	**− 2.06**	**22.83**	**0.05**
Cholesterol (%)	0.34 ± 0.03	0.34 ± 0.03	0.31	25.47	0.76
Glycogen + free glucose (%)	**1.02 ± 0.22**	**1.31 ± 0.22**	**− 3.45**	**24.74**	**0.00**
Energy (kJ/g)	26.70 ± 0.18	26.73 ± 0.28	− 0.34	20.34	0.74

	Nutritional history		Statistical analyses		
	Control (n = 11)	HC *(n* = *12)*	t	df	*p*-value
**Absolute content in egg**					
Mean egg mass (g)	0.07 ± 0.01	0.06 ± 0.01	1.25	17.24	0.23
Mean dry egg mass (g)	0.02 ± 0.00	0.02 ± 0.00	0.54	21.02	0.59
Protein content (mg per egg)	17.90 ± 3.12	17.01 ± 2.49	0.76	19.13	0.46
Lipid content (mg per egg)	6.71 ± 1.19	6.59 ± 0.98	0.26	19.51	0.80
Glycogen + free glucose (mg per egg)	0.68 ± 0.19	0.83 ± 0.18	− 1.93	20.72	0.07
Energy (kJ per egg)	1.87 ± 0.34	1.71 ± 0.26	1.26	18.46	0.22
Cortisol (ng per egg)	1.15 ± 0.43	1.01 ± 0.50	0.80	24.86	0.43

Surface, coefficient of variation of the surface among each spawn, average mass, average dry mass and biochemical composition of ’control eggs’ (i.e. eggs produced by females fed the control diet) and ’HC eggs’ (i.e. eggs produced by females fed the high carbohydrate diet). In bold, the parameters for which a significant difference was detected between the two conditions. (DM, dry matter).

The average mass (wet and dry) of ’control’ and ’HC eggs’ were not significantly different (Table 1). ’HC eggs’ had a significantly higher percentage of dry matter, lipid and total glucose (including glycogen and free glucose) than ’control eggs’ (Table 1). In term of absolute content, ’HC’ and ’control eggs’ had, on average, the same protein and lipid amount (Table 1). Finally, the cortisol content did not differ between control and ’HC eggs’ (p-value = 0.43, Table 1).

Eggs fatty acid profile differed between ’HC’ and ’control eggs’ (Table 2). While ’HC eggs’ had a higher proportion of saturated (+ 2.4%, p-value = 0.05) and mono-unsaturated FA (+ 8%, p-value < 0.001), they had a significantly lower proportion of omega 3 polyunsaturated fatty acids (PUFA) (− 10.5%, p-value < 0.001). More precisely, alpha-linolenic acid (ALA, 18:3 n-3), eicosapentaenoic acid (EPA, 20:5 n-3) and docosahexaenoic acid (DHA, 22:6 n-3) proportions were significantly lower in ’HC eggs’ in comparison to control ones. Even though no differences were recorded regarding omega 6 PUFA, linoleic acid (LA, 18:2 n-6) proportion was significantly lowered (− 12.5%, p-value = 0.05) and arachidonic acid (AA, 20:4 n-6) proportion was significantly increased (+ 8.6%, p-value < 0.05). The absolute content of saturated, mono-unsaturated and omega 6 PUFA were finally not significantly different between ’HC’ and ’control eggs’. Omega 3 PUFA content was significantly decreased in ’HC eggs’ (− 12.4%, p-value = 0.05). While EPA content was significantly decreased in ’HC eggs’ (− 22.8%, p-value<0.001), DHA content was not significantly different. Finally, while AA/EPA, AA/DHA ratios were significantly increased, EPA/DHA was significantly decreased in ’HC eggs’ (p-values < 0.01).

**Table 2:**
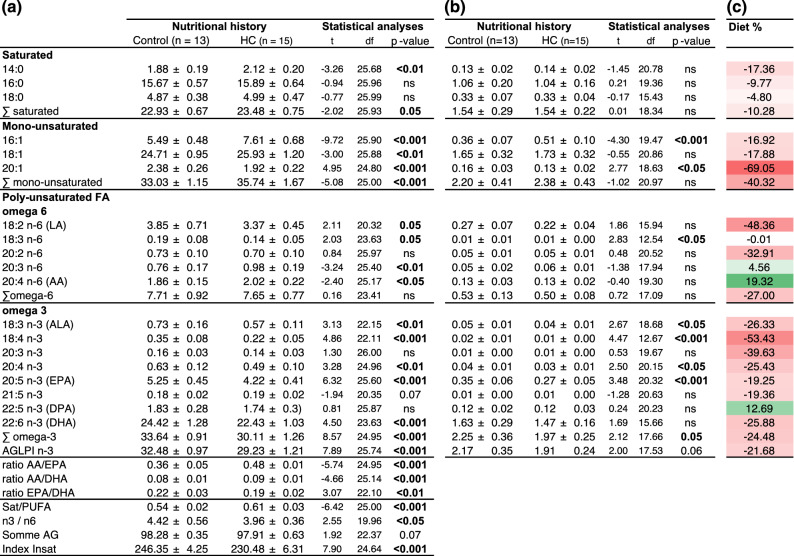
Major eggs fatty acid profile.

Fatty acid composition of ’control eggs’ (i.e. eggs produced by females fed the control diet) and ’HC eggs’ (i.e. eggs produced by females fed the high carbohydrate diet), expressed (a) either as a percentage of total FA (%), (b) or as an absolute quantity per eggs (mg/eggs). In bold, the fatty acids for which a significant difference was detected between the two conditions. (c) Difference in fatty acid composition between the control diet and the ’HC’ diet. Data are expressed as a percentage (%), with in green the fatty acids present in higher quantity in the HC diet and in red those present in lesser quantity in the HC diet.

### Embryo development

Six spawns per condition were fertilized with milts sampled from six males (n = 6 6 crossing per condition). Embryos development was daily monitored. Fertilization did not succeed for one of the ’control spawn’, whatever the milts used and was removed from the statistical analyses. While fertilization, hatching and survival (at both eyed stage and at hatching) rates were highly different from one female to another (p-values < 0.01), these parameters were not influenced by male identification. On average, fertilization rates of ’HC eggs’ tended to be lowered compared to the ones of the control ones (− 6.2%, p-value = 0.06). The survival of ’HC eggs’ were significantly decreased at eyed stage (− 9.9%, p-value = 0.03). Their hatching rates were however similar to those of the control ones. Finally, the survival of ’HC embryo’ at hatching tended to be reduced in comparison to the control ones (− 8.4%, p-value = 0.07).

The fertilization rate, the survival at eyed-stage and at hatching were negatively significantly correlated with AA proportion (%), AA/EPA and AA/DHA ratio in eggs and positively correlated with EPA proportion (%) and EPA absolute content (Supplementary Fig. S1).

Rates of malformation were not significantly different between the control and the ’HC fry’ (1.40 1.39% versus 1.32 1.39%, Table 3).

**Table 3:** Embryos development.

	Nutritional history		Statistical analyses		
	Control *(n* = *30)*	HC *(n* = *36)*	F	df	*p*-value
Fertilization rate (%)	90.8 ± 6.1	85.1 ± 6.3	4.48	9.00	0.06
**Survival at eyed stage (%)**	**83.7 ± 6.2**	**75.4 ± 7.4**	**6.26**	**9.00**	**0.03**
Hatchability rate (%)	91.8 ± 5.8	93.1 ± 3.5			*ns*
Survival at hatching (%)	76.7 ± 6.5	70.3 ± 7.6	4.29	9.00	0.07
Malformation rate (%)	1.40 ± 1.39	1.32 ± 1.38			*ns*

Fertilization, survival (at both eyed stages and at hatching) and hatching rates were monitored for ’control embryo’ (5 × 6 crossings) and ’HC embryo’ (6 × 6 crossings). In bold, the parameters for which a significant difference was detected between the two conditions.

### Fry development

After hatching, yolk-sac fry development and growth were assessed thanks to a morphological analysis. On average, the size of the fry born from females fed the HC diet (namely ’HC fry’) and the size of their yolk-sac were similar to the ones of the control fry (Table 4). However, the relative eye diameter was significantly higher in the ’HC fry’ (+ 4.2%, p-value = 0.05).

**Table 4:** Fry development.

	Condition		Statistical analyses		
	'Control fry'	'HC fry'	t	df	*p*-value
**Size**					
Length (cm)	1.72 ± 0.08	1.70 ± 0.06	0.67	20.79	*ns*
Height (cm)	0.51 ± 0.06	0.53 ± 0.05	− 1.00	19.68	*ns*
Whole body area (cm^2^)	0.50 ± 0.05	0.48 ± 0.05	1.04	20.96	*ns*
**Head morphology**					
Head length (cm)	0.35 ± 0.03	0.33 ± 0.02	1.77	20.33	0.09
Relative head length (%)	20.15 ± 1.38	19.36 ± 0.97	1.58	18.11	0.13
Eye diameter (cm)	0.14 ± 0.01	0.15 ± 0.01	− 1.48	18.81	0.16
Relative eye diameter (%)	8.26 ± 0.29	8.61 ± 0.50	− 2.09	17.92	**0.05**
**Yolk sac morphology**					
Yolk sac length (cm)	0.71 ± 0.06	0.69 ± 0.05	0.64	20.54	*ns*
Relative yolk sac length (%)	40.75 ± 3.14	40.85 ± 2.47	− 0.09	18.97	*ns*
Yolk sac height (cm)	0.41 ± 0.06	0.42 ± 0.03	− 0.55	16.26	*ns*
Relative yolk sac height (%)	79.62 ± 6.02	78.29 ± 4.37	0.68	16.97	*ns*
Yolk sac area (cm^2^)	0.23 ± 0.04	0.22 ± 0.03	0.96	20.90	*ns*
Relative yolk sac area (%)	46.08 ± 4.27	45.37 ± 3.95	0.41	20.42	*ns*
Volume yolk sac (cm^3^)	0.06 ± 0.02	0.06 ± 0.01	− 0.10	16.91	*ns*

Whole body morphologies, head morphologies and yolk-sac morphologies were assessed for fry born from the ’control females’ and fry born from females fed the high carbohydrate diet (’HC’). In bold, the parameters for which a significant difference was detected between the two conditions.

## Discussion

In the future years, the traditionally protein-rich aquafeed must evolve in aquaculture practice, and a possible incorporation of plant-derived carbohydrates will lower the protein-to-carbohydrate ratio^7^. It is now acknowledged that maternal nutrition has drastic outcomes on their progeny^11^ and the consequences of high carbohydrate diet in fish must be explored.

First, female trout broodstock can grow normally throughout an entire reproductive cycle when fed a diet in which more than 30% of the traditionally included fishmeal is replaced by plant-derived carbohydrates. We have further confirmed here that the glucose intolerance phenotypes attributed to teleost fish species belonging to higher trophic levels^12^ seems in fact highly dependent on their life stage and that female broodstock trout are better able to use such nutrient than juveniles^13^. The aquaculture sector could take advantage of these differences to improve the sustainability of their broodstock aquafeed.

Regarding reproductive performances which include the total number of eggs and proportion of viable eggs within spawns, the results were more contrasted. In one hand, whatever the diet given to the females, the number of viable eggs within spawn was high and corresponds to what is generally found under normal rearing conditions^14^. Only one spawn produced by a female fed a high carbohydrate nutrition, had a high proportion of white eggs, i.e. eggs with a weak vitelline membrane that breaks during the egg water-hardening step^10^. One year high carbohydrate nutrition had even increased the relative fecundity (+ 24.1%), confirming results obtained in one of the previous studies investigating the effects of such nutrition in trout^8^. Such augmentation may in fact hide a decrease in average egg size, as a trade-off exist between the number of eggs produced and the eggs size (i.e. fish with lower fecundity typically produce larger eggs and vice versa)^15^. In order to evaluate the quality of a spawn, the joint consideration of these last two parameters is therefore essential. However, in the present study, the size of the produced eggs was, in average, not affected by the maternal high carbohydrate nutrition, in accordance with previous results obtained^9^.

Nevertheless, in the other hand, the size of the fish and spawn weight, known to be highly correlated in trout^21^, were not correlated when female were fed the high carbohydrate diet. Therefore, different strategies to cope with the high carbohydrate nutrition seem to have been implemented by females. More precisely, among the population, one heavier and longer female has produced less eggs than expected and seems to have therefore selected the body maintenance over their reproduction (Fig. 1). Interestingly, most of the sampled females seem to have sacrificed somatic tissues to ensure the production of the eggs, and had therefore a high relative fecundity. However, for these ones, even though the average eggs size remained unaffected, the intra-spawn variability in eggs size was particularly high (Fig. 1), indicating a disruption of vitellogenesis induced by the high carbohydrate nutrition. Only one female fed the high carbohydrate diet has produced a spawn of quality (low variability in eggs size) but has displayed the lowest relative fecundity (Fig. 1).

Vitellogenesis is a multifactorial and complex process, and several hypotheses can be considered to explain the disruption observed for some female fed the high carbohydrate diet. First, it could be hypothesized that such nutrition does not fully meet the female nutritional requirements. The mobilization of nutrient reserves might be insufficient to allow the correct development of all eggs. Second, dietary carbohydrates modulate the level of insulin-like growth factor-I in fish^16^, which, in turn, is known to affect the production of reproductive hormones^17,18^. Therefore, it could be also hypothesised that one year high carbohydrate nutrition might impact the vitellogenesis process by misregulating hormonal system. Finally, in rainbow trout, a stress applied during vitellogenesis is known to increase heterogeneity within spawns, due to the maintenance of some eggs and the atresia of the others^19,20^. A stress, other than nutritional, might also impact the correct development of the entire spawn. Both the variability of the strategies established by the females and the variability within the same spawn regarding eggs size could be considered as flaws for aquaculture production that seeks to maximize the homogeneity in their population.

Because egg matter composition is one of the most important factors determining the proper development of the embryo^4^, it is essential to also consider this parameter when assessing maternal high carbohydrate nutrition. Such maternal nutrition increased lipid, total glucose content, and in lesser extent protein, in eggs, due to the increased of dry matter. The absolute content (in mg per eggs), which is available for the embryo to develop, remained in average unchanged by the maternal nutrition suggesting that one year high carbohydrate nutrition allows the production of eggs whose biochemical quality is maintained as previously demonstrated^8,9^. However, as yolk is the major component of the egg^22^, the variation observed in ’HC eggs’ size could therefore indicate a variation in eggs biochemical composition within spawns and in the amount of energy available for embryo development. Such a heterogeneity would therefore also be a weakness for the production.

A particular importance should also be paid to lipids and more particularly to both the absolute amount of essential FA and their balance. First, FA are a major energy source for embryo development. Docosahexaenoic acid (DHA) and eicosapentaenoic acid (EPA) also play an important structural role as key components of cell membranes and ensure their correct physiological functions^23^. In addition to having an essential structural role, EPA and arachidonic acid (AA) are the precursors of eicosanoids (prostaglandin and leukotriene), which have been demonstrated to have an action on final maturation, ovulation and spawning in sea bass^24^. Those three FA are necessary to ensure a proper reproduction (i.e. oocyte maturation, ovulation, spawning and fertilization) and the correct development of the embryo, as demonstrated in various fish species^5,25–32^.

**Figure 2:**
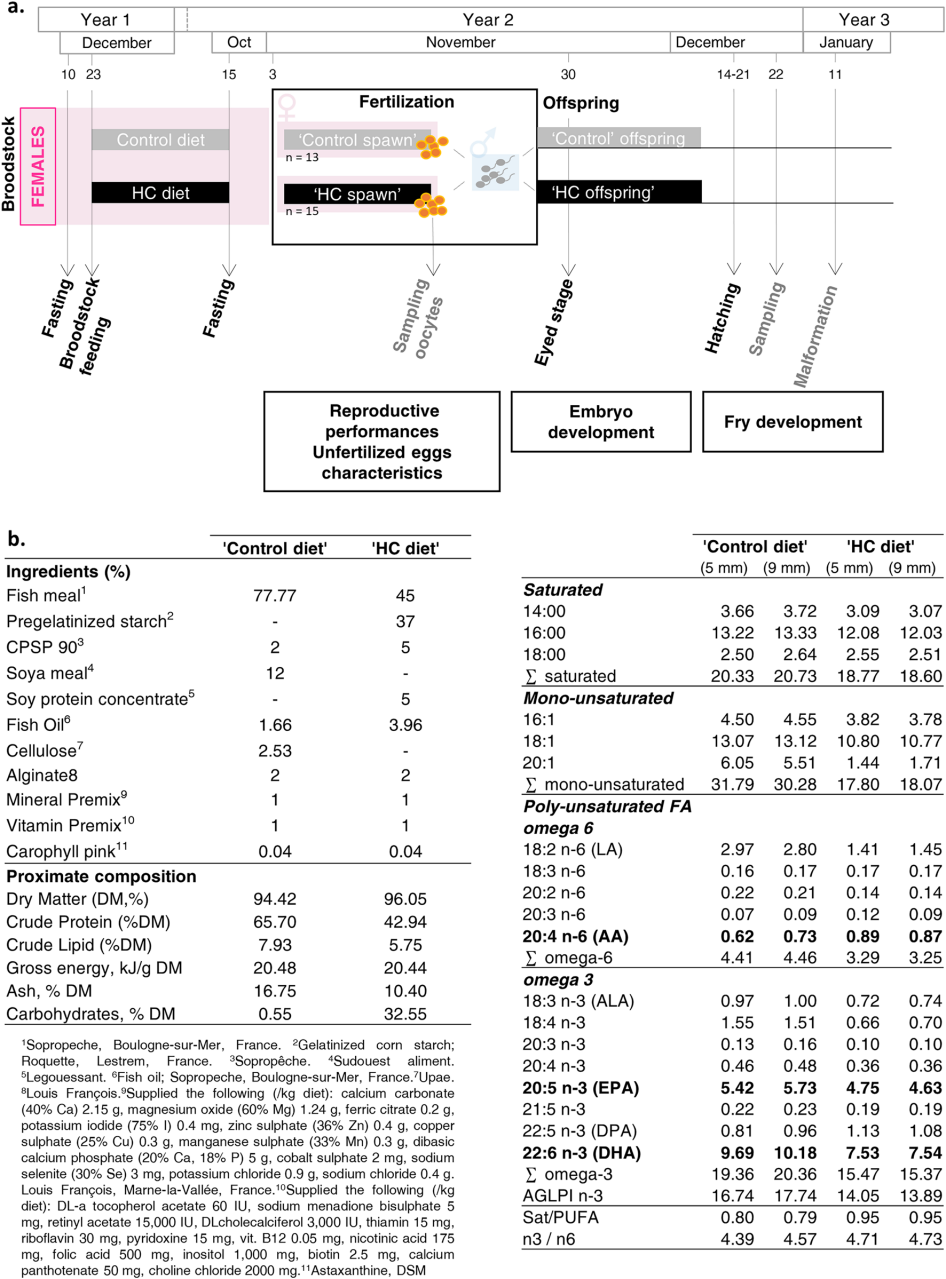
(**a**) Experimental design. (**b**) diet composition and fatty acid profile (in mg/g diet). Broodstock females were fasted after spawn from 10 to 23 December. Then, they were fed for one year either the control diet or a diet formulated with plant-derived carbohydrates, a high carbohydrate diet (’HC diet’). From the 15 of October to the 3 of November, fish were fasted again. Control and ’HC’ spawns were sampled in order to assess the reproductive performances and the quality of the unfertilized eggs. Fertilizations were carried out to obtained control progeny and ’HC progeny. The development of the progeny were carefully monitored until the first feeding.

Fatty acid profile differed between the diet formulated with fishmeal and the one formulated with plant-derived carbohydrates, especially regarding the absolute content in AA which is 19.3% higher and EPA which is 19.3% lower (Fig. 2b and Table 2), due to the different inclusion of fish oil. Eggs fatty acid profile is known to partly reflect the maternal diet and 74% of the studied FA contained in the eggs had their proportion affected (Table 2). In line with the maternal diet composition, an increase in AA proportion and AA/EPA ratios and a decrease in 20:1 proportion were the most notable changes observed. More importantly, the one year high carbohydrate nutrition significantly decreased both DHA and EPA proportions and EPA content in eggs. DHA and, in a lesser extent EPA, are normally selectively mobilized and incorporated into the eggs during vitellogenesis at the expense of other tissue composition if they are lacking in the diet^33,34^. 1 year high carbohydrate nutrition is therefore too drastic to allow the sufficient mobilization of both DHA and EPA to reach their expected levels in eggs.

Fertilization and survival at eyed-stage rate, and to a lesser extent at hatching, were correlated to some extent with AA (negative correlation) and EPA (positive correlation) levels, but not DHA. Although these results need to be confirmed with a larger number of samples, it suggests that both the increase in AA and the decrease in EPA in eggs are part of the factors causing the decrease in fertilization and survival at eyed-stage when females were fed the high carbohydrate nutrition. The importance of these two fatty acids for the correct development of the embryo has already been demonstrated in gilthead sea bream^25,26^ and in european sea bass^27^. As described above, the reserves of the females fed the high carbohydrate nutrition were not uniformly allocated to the eggs. Even though the size of the eggs do not correlate with the survival rate in trout^35,36^, it could not be excluded that smaller eggs could have not store enough nutrient and are thus unable to develop properly, reducing thus the average survival rate. This hypothesis would explain why no difference was found later in the growth and morphology of the fry.

## Conclusion

Decreasing of the protein-to-carbohydrate ratio in aquafeed for fish female broodstock belonging to high trophic level has, in average, not impaired fish growth and has no drastic negative consequences on the quality of produced spawns. Replacement of traditional protein-rich fishmeal by plant-derived carbohydrates, available in larger quantities and at lower cost, is therefore feasible for trout. Nevertheless, the replacement of 30% of the protein supply by fishmeal increased the variability of eggs size within spawns. Some eggs were smaller than others and had, in average, a modified fatty acid profile, probably less suitable for embryonic development. These two elements could explain the observed reduced survival of their progeny. Adjustment of feed formulation is needed (e.g. diminution of carbohydrate proportion to 20%), before it could be applied in aquaculture practice. The remaining eggs produced quality offspring, whose growth and morphology were not impaired in the short term.

## Methods

### Broodstock breeding and experimental design

Three-year old female rainbow trout (*Oncorhynchus mykiss*) were distributed into two 8 m^3^ tanks at an initial density of 22 kg m^3^. The experiment was conducted at the INRAE Lées-Athas experimental fish farm facility and reared under a natural photoperiod at 8 °C. Fish were first fasted for 13 days after the second reproductive event and were then fed with either a control diet devoid of carbohydrate (0.6% carbohydrate) or an experimental high carbohydrate (HC, 32.5%) diet (Fig. 2a).

The two experimental diets have been previously described by Favalier et al.^13^. These two diets were formulated to be isoenergetic and were prepared in our own facilities (INRAE, Donzacq, France) as extruded pellets (9 mm pellets, BC45 BisVis Clextral, France). In both diets, fish oil was included as the lipid sources. In the control diet, fishmeal was used as the protein source. In the HC diet, fishmeal was also used as the protein source and carbohydrates were provided as gelatinized starch (Fig. 2b). Fish were fed at 1% of body weight (at this percent all diet were consumed in the rearing conditions applied here) to ensure that the feed intake was the same between tanks. The feeding experiment lasted 11 months, until the next third reproduction.

### Sampling and fertilization

During the spawning period, fish feeding was stopped and fish were closely monitored in order to detect ovulating females. At the spawning peak (17 days after the detection of first ovulation), 13 control ovulating females and 15 ovulating females fed the HC diet were sampled. To do that, females were sedated by immersion in MS-222 solution (50 mg/L) and were then measured (in cm), weighed, (in g) and manually stripped. Whole spawn (eggs and coelomic fluid) and eggs alone were weighed. Approximatively 20 g of unfertilized eggs (per female) were sampled to assess the quality of the reproductive performances (details below). Moreover, in order to characterize eggs composition, 8 g of unfertilized eggs (per female) were also sampled and stored at − 20 °C until further analyses (details below).

Before fertilization, six 3-year old male trout, fed during 11 months with the control diet, were stripped. Two ml of milt (per male) were pre-diluted in the StorFish solution (IMV technologies, L’Aigle, France) and stored at 4 °C until utilization. Among the previously sampled females, six control females and six females fed the HC diet were used for both pool fertilization and individual fertilization (Supplementary Fig. S2). For individual fertilization, eggs recovered for each female (9 g per female) were equally distributed into 6 batches (36 control batches and 36 ’HC batches’). Each batch was individually cross-fertilized with 500 µL of the diluted milts previously collected and blended with the Actifish solution (sperm motility activating saline solution, IMV technologies, L’Aigle, France). After the removing of the Actifish solution, the 72 batches containing fertilized egg were transferred into individual incubators. For pool fertilization, eggs recovered (10 g per female) were fertilized with a pool of diluted milt (1 mL of diluted milt per male) and were then blended with the Actifish solution. After the removing of the Actifish solution, the two batches of fertilized eggs (control and HC batches) were transferred into two large incubators. Incubators were maintained at the INRAE Lées-Athas experimental fish farm facility at 8 °C and were daily monitored.

### Reproductive performances: spawn characteristics

In order to estimate number of eggs per spawn, proportion of non-viable eggs (white eggs) per spawn and size of the eggs, approximatively 20 g of unfertilized eggs sampled were precisely weighed and stored in 150 mL containers with water in order for the eggs to hydrate. After 24 h, pictures of hydrated eggs were performed and processed using the VisEGG tool, which allow a rapid and automatic measurement of different spawns variables (number and size of the eggs and white egg percentage)^10^. Absolute fecundity (per female), i.e., total number of eggs produced by female, was automatically estimated by VisEGG. Relative fecundity (per female) was calculated as follows:

1 $${\text{Relative\;fecundity }} = {\text{Absolute\;fecundity}}\;/\;{\text{Female\;mass}}$$

### Eggs biochemical composition

Eggs biochemical composition was determined, as described in Callet et al.^9^. Briefly, eggs were counted, crushed, weighted and then freeze dried. After the lyophilization process, eggs were weighed again to calculate average dry matter content (DM, in %) and average dry eggs mass (in g). The gross energy was measured on dried eggs in an adiabatic bomb calorimeter (IKA, Heitersheim Gribeimer, Germany). Crude protein (N × 6.25) was determined on dried eggs by the Kjeldahl method after acid digestion. Average eggs protein content was estimated as follows:

2 $${\text{Protein}}\;{\text{ content }}\left( \% \right)\; = \;{\text{Protein}}\;{\text{ content}}\left( {\% {\text{DM}}} \right)\;\times\;{\text{DM }}\;{\text{content}}\left( \% \right)/\;{1}00$$

3 $${\text{Protein}}\;{\text{ content }}\left( {{\text{mg}}\;{\text{ per}}\;{\text{ egg}}} \right)\; \, = \, \;{\text{Protein }}\;{\text{content}}\left( \% \right)\;/\;{1}00 \, \; \times \, \;{\text{Egg}}\;{\text{ mass }}\; \times \;{ 1}000$$

The total glucose content was determined by a hydrolysis technique previously described by Good et al.^37^. Total lipid content was determined using dichloromethane/methanol (2:1, v/v), according to Folch et al.^38^. Lipid content was estimated as follows:

4 $${\text{Lipid}}\;{\text{ content }}\left( {{\text{mg }}\;{\text{per }}\;{\text{egg}}} \right)\; \, = \;{\text{Lipid }}\;{\text{content}}\left( \% \right)\;/\;{1}00 \, \; \times \;{\text{ Egg}}\;{\text{ mass}}\; \, \times \;{ 1}000$$

In order to analyze egg fatty acid (FA) profile, fatty acid methyl esters (FAME) were prepared by acid-catalyzed transmethylation, using boron trifluoride according to Shantha and Ackman^39^. FAME were then analyzed in a Varian gas chromatograph and FA were identified with reference to a known standard mixture (Sigma, St Louis, MO, USA) and peaks were integrated using Varian Star Chromatography Software (Star Software, version 5). The results for individual FA were expressed as percentage of total identified FAME and FA contents were estimated as described in Callet et al.^9^

5 $${\text{FA}}\; \, \left( {{\text{mg }}\;{\text{per}}\;{\text{ egg}}} \right)\; \, = \;{\text{FA }}\;{\text{content}}\left( \% \right)\;/\;{1}00 \, \; \times \;{\text{ Lipid }}\;{\text{content }}\;\left( {{\text{mg }}\;{\text{per }}\;{\text{egg}}} \right)$$

### Embryo and yolk-sac fry development

Dead embryos were daily removed and recorded. At eyed-stage, embryos held in individual incubators were counted. The exact number of fertilized eggs were then determined by adding the count of eyed-stage embryos to the number dead embryos in the course of the experiment. Fertilization rates were then estimated per female as follows:

6 $${\text{Fertilization }}\;{\text{rate}}\; \, \left( \% \right)\; \, = \;{ 1}00\; \, \times \;{\text{Total}}\;{\text{number}}\;{\text{of}}\;{\text{embryo}}\left( {{\text{J}}\; + \;{1}} \right)\;/\;{\text{Total}}\;{\text{number}}\;{\text{of}}\;{\text{fertilized}}\;{\text{eggs}}$$

The survival at eyed-stage was estimated per female, as follows:

7 $${\text{Survival}}_{{{\text{ eyed}} {\text{ stage}}}} \left( \% \right)\; \, = \;{ 1}00\; \, \times \;{\text{Total }}\;{\text{number }}\;{\text{of }}\;{\text{eyed }} \; {\text{stage }}\;{\text{embryo }}/{\text{ Total }}\;{\text{number }}\;{\text{of }}\;{\text{fertilized }}\;{\text{eggs}}$$

Just after hatching, yolk-sac fry held in individual incubators were counted and survival and hatchability were estimated per female, as follows:

8 $${\text{Survival}}_{{{\text{ hatching}}}} \left( \% \right)\; \, = \;{ 1}00 \, \times {\text{Total}}\;{\text{number}}\;{\text{of}}\;{\text{yolk}}\;{\text{sac }}\;{\text{fry}}\;/\;{\text{Total}}\;{\text{number}}\;{\text{of}}\;{\text{fertilized}}\;{\text{eggs}}$$

9 $${\text{Hatching }}\;{\text{rate }}\left( \% \right) \, \; = \, \;{1}00\; \times \;{\text{Total}}\;{\text{number}}\;{\text{of}}\;{\text{yolk}}\;{\text{sac}}\;{\text{fry}}\;/\;{\text{Total}}\;{\text{number}}\;{\text{of}}\;{\text{eyed}}\; {\text{stage}}\;{\text{embryo}}$$

Finally, 12 yolk-sac fry per condition were sampled in the pool incubators, anesthetized in a benzocaine bath (30 mg/L, n = 12 per condition) and then euthanized in a benzocaine bath at a concentration of 60 mg/L. A picture of each yolk-sac fry was taken in order to characterize fry body morphologies.

Body and yolk-sac measurements were performed for each fry by two different people using the software ImageJ (https://imagej.nih.gov/ij/index accessed on 1 March 2020), as previously described by Callet et al.^40^. To characterize fry body morphologies, the fork length (L), the body depth (D), the head length (HL), the eye diameter (ED) and the whole-body area (WBA) were measured. The relative head length (HLr) and relative eye diameter (EDr) were calculated by dividing HL and ED by fry length. To describe fry yolk-sac morphologies, the yolk-sac length (YSL), height (YSH), and area (YSA) were measured. Then, the relative yolk-sac area (YSAr), height (YSHr), and length (YSLr) were calculated by dividing YSA, YSH, and YSL by WBA, D, and L, respectively. The yolk-sac volume (YSV) was also estimated as follows:

10 $${\text{YSV }}\;\left( {{\text{mm}}^{{2}} } \right) \, \; = \, \;\pi /{6}\; \, \times \, \;{\text{YSL}}\; \, \times \;{\text{ Y SH}}^{{2}}$$

Malformation rates (spinal cord torsion, head or caudal fin malformations) were recorded in yolk-sac fry from individual incubators at the end of the trial.

### Statistical analyses

All the statistical analyses were performed using R Software (version 3.2.5). Either linear mixed-effects models or Student’s T-tests were used to test the effects of the female broodstock nutritional history on the different parameters recorded (with a significance threshold set at p-value = 0.05). An arcsine transformation on proportion data was first applied. Concerning female zootechnical performances, spawn characteristics and eggs biochemical composition Student’s T-tests were used. Concerning egg size, obtained with VisEGG, the package ’lme4’ from R software was used to run linear mixed models. Female identification was treated as random effects to take into account the intra-female variability (biological replicate, approximatively 200 eggs measured/female). The best model was then selected using the Akaike Information Criterion. Parameters regarding embryo/yolk-sac fry development were monitored in batches obtained from individual fertilization (n = 6 batch/female and n = 6 batch/male). The effect of the female broodstock nutritional history was assessed using linear mixed models and the female and male identification were then treated as random effects to take into account both the intra-female and intra-male variability (biological replicate) and the variability between batches (technical replicates).Yolk-sac fry morphologies were measured in fry obtained from pooled fertilization and Student’s T-tests were used to assess the effect of female broodstock nutritional history. Finally, correlations were estimated using a Pearson test (cut-off *p* = 0.05).

### Ethical statement

Investigations were conducted according to the guiding principles for the use and care of laboratory animals and in compliance with French and European regulations on animal welfare (Décret 2001-464, 29 May 2001 and Directive 2010/63/EU, respectively). This protocol and the project as a whole were approved by the French National Consultative Ethics Committee (reference numbers 2019090210165534-v3). All methods are reported in accordance with ARRIVE guidelines.

## Supplementary Information

Supplementary Figures.

## Data Availability

The datasets used and/or analysed during the current study available from the corresponding author on reasonable request.
