# Supplementary material for: Alteration of eggs biochemical composition and progeny survival by maternal high carbohydrate nutrition in a teleost fish
Source: Sci Rep. 2022 Oct 6;12:16726. doi: 10.1038/s41598-022-21185-5 (PMC9537176; doi:10.1038/s41598-022-21185-5)
Supplement: Supplementary file 1 — Supplementary Figures. [file 41598_2022_21185_MOESM1_ESM.docx]

A maternal high carbohydrate and low protein nutrition have altered eggs biochemical composition and progeny survival in a teleost fish, the rainbow trout,

Callet *et al.,*

**Supplementary Figure S1**


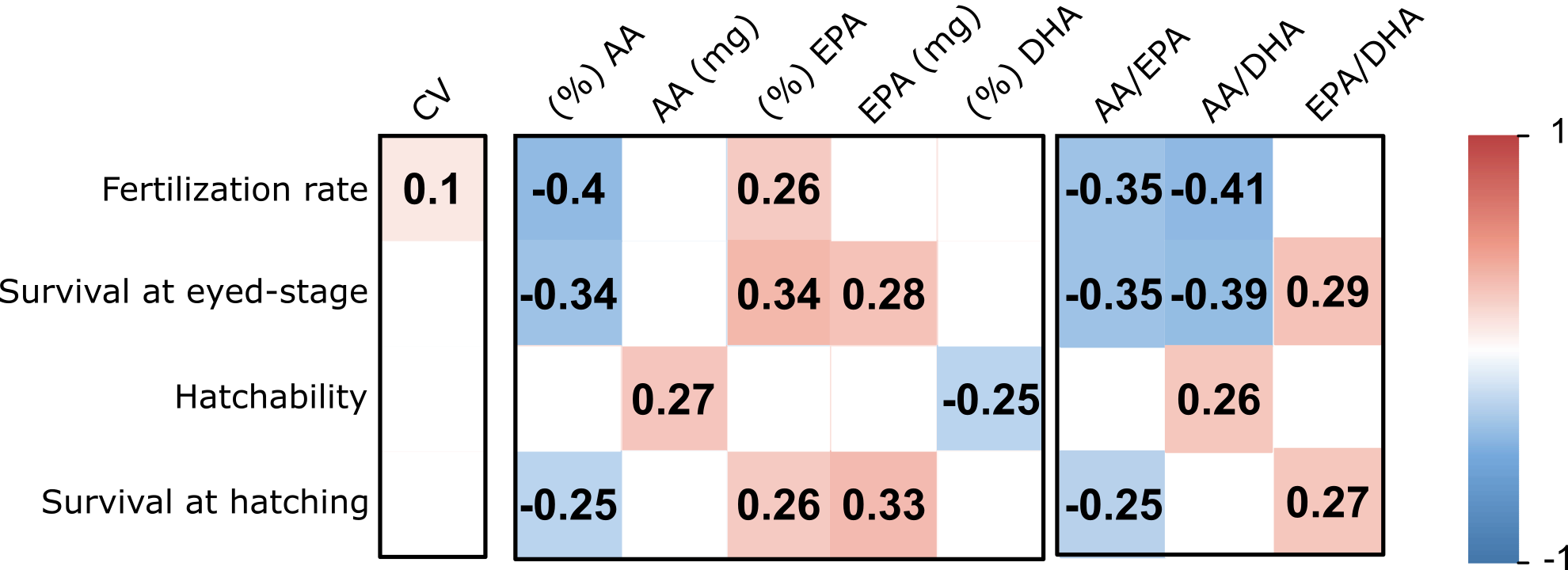


Matrix of correlation between eggs characteristics (Coefficient of variation ‘CV” of eggs surface, arachidonic acid (AA), eicosapentaenoic acid (EPA) docosahexaenoic acid (DHA) proportions and absolute contents, and their ratio in eggs) and embryo development parameters. Correlation were investigated using Pearson tests (cut-off p = 0.05), and each value is indicated inside the matrix, with significant positive correlations in red and significant negative ones in blue.

**Supplementary Figure S2**


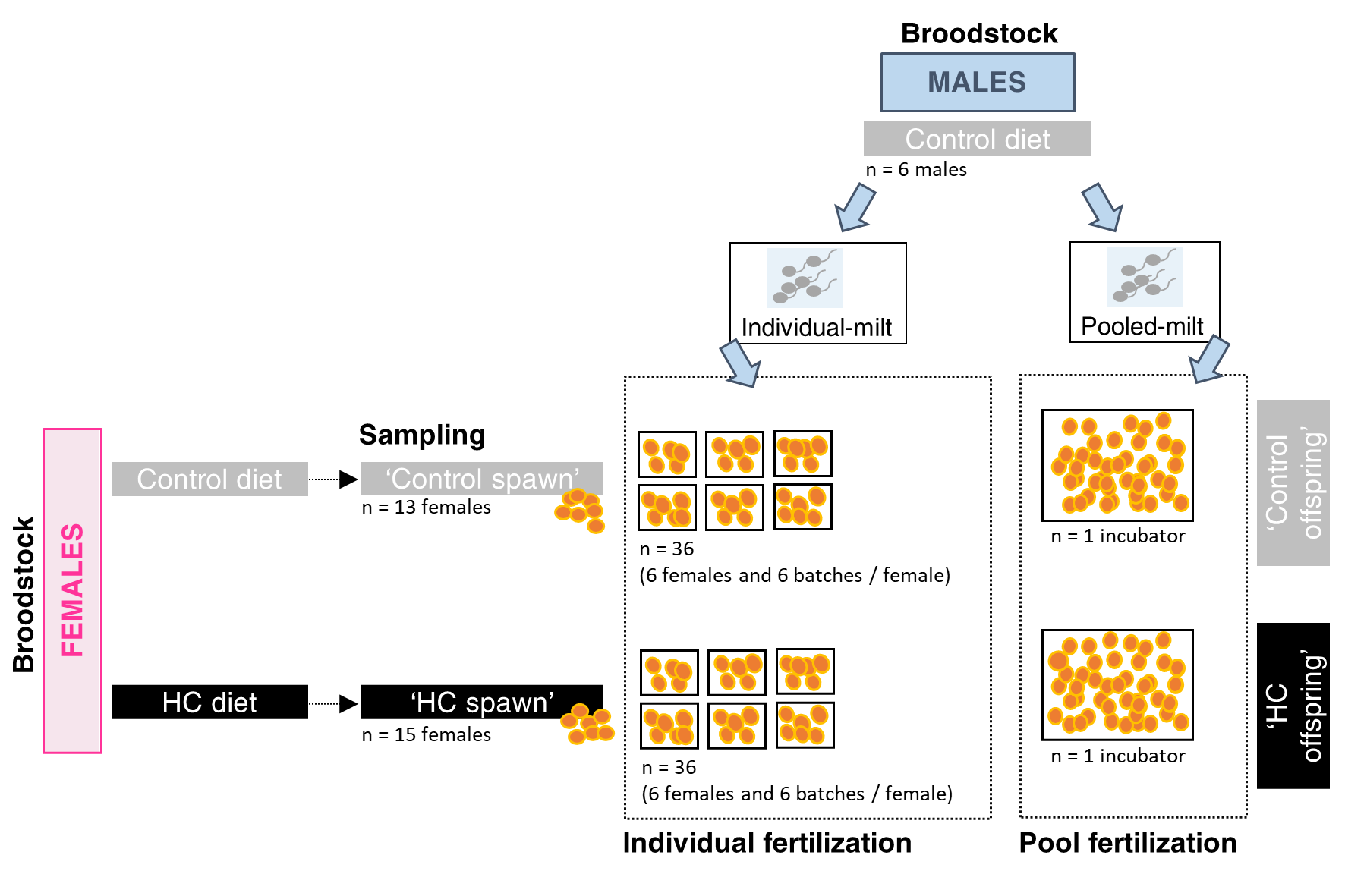


Six three-year old male trout, fed during 11 months with the control diet, were stripped to collect milts. At the same time, 13 control ovulating females and 15 ovulating females fed the high carbohydrates diet (‘HC diet’) were sampled. Among these sampled females, six control females and six females fed the HC diet were used for both pool fertilization and individual fertilization. For individual fertilization, recovered eggs were equally distributed into 72 batches (6 batches / female) and cross-fertilized with 500 µL of the diluted milts previously collected. For pool fertilization, a pool of the eggs recovered (10 g per female) were fertilized with a pool of diluted milt. The two batches (control and ‘HC’ batches) of fertilized eggs were transferred into two large incubators.
